# Eosinophils and eosinophil-associated disorders: immunological, clinical, and molecular complexity

**DOI:** 10.1007/s00281-021-00863-y

**Published:** 2021-05-30

**Authors:** Peter Valent, Lina Degenfeld-Schonburg, Irina Sadovnik, Hans-Peter Horny, Michel Arock, Hans-Uwe Simon, Andreas Reiter, Bruce S. Bochner

**Affiliations:** 1 Department of Internal Medicine I, Division of Hematology and Hemostaseology, Medical University of Vienna, Währinger Gürtel, 18-20 1090 Vienna, Austria; 2 Ludwig Boltzmann Institute for Hematology and Oncology, Medical University of Vienna, Vienna, Austria; 3 Institute of Pathology, Ludwig Maximilian University, Munich, Germany; 4 Laboratory of Hematology, Pitié-Salpêtrière Hospital, Paris, France; 5 Institute of Pharmacology, University of Bern, Bern, Switzerland; 6 Department of Clinical Immunology and Allergology, Sechenov University, Moscow, Russia; 7 Laboratory of Molecular Immunology, Institute of Fundamental Medicine and Biology, Kazan Federal University, Kazan, Russia; 8 Department of Hematology and Oncology, University Hospital Mannheim, Heidelberg University, Mannheim, Germany; 9 Division of Allergy and Immunology, Department of Medicine, Northwestern University Feinberg School of Medicine, Chicago, IL USA

**Keywords:** Hypereosinophilia, Eosinophilic leukemia, *FIP1L1-PDGFRA*, Hypereosinophilic Syndromes, Classification, Targeted therapy

## Abstract

Eosinophils and their mediators play a crucial role in various reactive states such as bacterial and viral infections, chronic inflammatory disorders, and certain hematologic malignancies. Depending on the underlying pathology, molecular defect(s), and the cytokine- and mediator-cascades involved, peripheral blood and tissue hypereosinophilia (HE) may develop and may lead to organ dysfunction or even organ damage which usually leads to the diagnosis of a HE syndrome (HES). In some of these patients, the etiology and impact of HE remain unclear. These patients are diagnosed with idiopathic HE. In other patients, HES is diagnosed but the etiology remains unknown — these patients are classified as idiopathic HES. For patients with HES, early therapeutic application of agents reducing eosinophil counts is usually effective in avoiding irreversible organ damage. Therefore, it is important to systematically explore various diagnostic markers and to correctly identify the disease elicitors and etiology. Depending on the presence and type of underlying disease, HES are classified into primary (clonal) HES, reactive HES, and idiopathic HES. In most of these patients, effective therapies can be administered. The current article provides an overview of the pathogenesis of eosinophil-associated disorders, with special emphasis on the molecular, immunological, and clinical complexity of HE and HES. In addition, diagnostic criteria and the classification of eosinophil disorders are reviewed in light of new developments in the field.

## Introduction

Eosinophil granulocytes are highly specialized hematopoietic effector cells that play a crucial rule in host defense and tissue remodeling. Eosinophils produce, store, and release an array of biologically active substances, including cytotoxic proteins, lipid mediators, chemotactic proteins (chemokines), and cytokines [1–4]. Under various physiologic conditions and pathologies, eosinophils migrate into certain target organs, and once activated, release their products in affected tissue sites, thereby promoting local inflammation, tissue remodeling, and sometimes tissue damage [1–9]. When peripheral blood eosinophilia persists and exceeds 1500 cells/μL blood, the term hypereosinophilia (HE) is appropriate [7–11]. In patients with HE, eosinophil-derived effector molecules can provoke substantial alterations in the tissue microenvironments [1–11]. In these cases, blood HE is usually accompanied by tissue HE with local accumulations of eosinophils in affected organs and deposition of eosinophil-derived cytotoxic and tissue-remodeling proteins. In a subset of these patients, tissue fibrosis and/or thrombosis with end organ damage develops, resulting in the diagnosis of a HE syndrome (HES) [6–11]. The organ dysfunction induced by HE may be reversible or irreversible, depending on the magnitude and duration of HE, the underlying etiology, the presence of certain co-morbidities, and response to therapy.

Blood and tissue HE can be observed not only in a variety of reactive conditions, including allergic or inflammatory diseases, auto-immune disorders, drug reactions, or infectious diseases, but also in certain hematologic neoplasms, and sometimes in patients with solid tumors (Table 1) [4, 6–19]. Persistent reactive HE is typically found in chronic (untreated) helminth infections, other chronic infections, auto-immune disease processes, other inflammatory reactions, and atopic disorders (Table 1).

**Table 1:** Conditions and disorders potentially associated with hypereosinophilia (HE)

Reactive non-neoplastic conditions - secondary/reactive HE (HE_R_)*	
Chronic infections: viral, bacterial, fungal (e.g., aspergillosis)	
Parasitosis (e.g., helminth infections)	
Infestations (e.g., scabies) Allergic or toxic drug reactions	
Intoxication: toxic oil syndrome, others	
Allergic disorders, including atopic dermatitis and allergic asthma	
Acute and chronic graft-versus-host disease	
Auto-immune disorders – rheumatologic disorders	
Chronic inflammatory disorders, including EGID	
Lymphoid variant of hypereosinophilic syndrome (HES-L)	
Myeloid neoplasms and stem cell neoplasms with HE (neoplastic/primary HE: HE_N_)**	
Hematopoietic neoplasms with eosinophilia and rearranged *PDGFRA*	
Hematopoietic neoplasms with eosinophilia and rearranged *PDGFRB*	
Hematopoietic neoplasms with eosinophilia and rearranged *FGFR1*	
Hematopoietic neoplasms with eosinophilia and *PCM1-JAK2*	
Chronic eosinophilic leukemia – NOS	
Chronic myeloid leukemia (CML-eo) – *BCR-ABL1*+	
Myeloproliferative neoplasms (MPN) with HE (MPN-eo)	
Systemic mastocytosis (SM) with HE (SM-eo)***	
Myelodysplastic syndrome (MDS) with HE (MDS-eo)	
MPN/MDS overlap syndromes with HE (MPN/MDS-eo; e.g., CMML-eo)	
AML with inv(16) and eosinophilia (AML-M4-eo)	
Neoplastic conditions with secondary/reactive HE (paraneoplastic HE_R_)*	
Solid tumors/cancers (lung, GI tract, others)	
Langerhans cell histiocytosis	
Hodgkin’s disease	
B or T cell non-Hodgkin’s lymphoma	
B or T cell leukemia	

^*^In these patient groups, eosinophilia is usually caused by cytokines that promote the growth and accumulation of eosinophils and their precursor cells

^**^In these disorders, eosinophils are usually derived from the neoplastic clone (from clonal stem cells)

^***^Eosinophilia or even HE develop quite frequently in patients with advanced systemic mastocytosis (SM), such as aggressive SM (ASM), but may also occur in indolent SM (ISM) or smoldering SM (SSM)

*EGI* eosinophil-associated gastrointestinal disorders, *NOS* not otherwise specified, *CML* chronic myeloid leukemia, *MDS* myelodysplastic syndrome, *MPN* myeloproliferative neoplasm, *CMML* chronic myelomonocytic leukemia, *AML* acute myeloid leukemia, *GI tract* gastrointestinal tract

Hematopoietic malignancies that may present with marked persistent HE encompass myeloproliferative neoplasms (MPN-eo), certain forms of acute myeloid leukemia (AML-eo), a small subset of patients with myelodysplastic syndromes (MDS-eo), some MDS/MPN overlap disorders, certain lymphoproliferative disorders, including T cell Non-Hodgkin’s lymphoma (NHL), and systemic mastocytosis (SM-eo) (Table 1) [10–19]. These differential diagnoses must be considered in patients with unexplained HE or HES, especially in the context of additional blood count abnormalities. Therefore, it is standard to perform hematologic investigations, including bone marrow (BM) examinations and molecular studies in such cases [13–19].

In patients suffering from a myeloid or stem cell–derived hematopoietic neoplasm, eosinophils usually belong to the neoplastic clone. In some of these patients, fusion genes involving platelet-derived growth factor receptor (*PDGFR)A*, *PDGFRB*, fibroblast growth factor receptor-1 (*FGFR)-1*, Janus kinase-2 (*JAK2*), or other target genes are detected (Table 1) [10, 11, 14–18]. This is important as PDGFR-targeting tyrosine kinase inhibitors (TKI), like imatinib, are effective in most *PDGFR*-rearranged malignancies, but not in those with other molecular abnormalities, such as *FGFR-1* mutations [10, 11, 14–18].

During the past decades, our knowledge on eosinophils, their products, eosinophil-rich neoplasms, and the mechanisms underlying HE and HES-specific organ damage has improved considerably [5–19]. Moreover, a number of proposals for the classification of HE, HES, and related syndromes have been published [7–11, 14–19]. The current article provides an update and review of current concepts around the diagnosis, classification, and management of HE, HES, and related eosinophil-rich neoplasms.

## Cytokine-mediated differentiation, migration, and activation of eosinophils

**Fig. 1:**
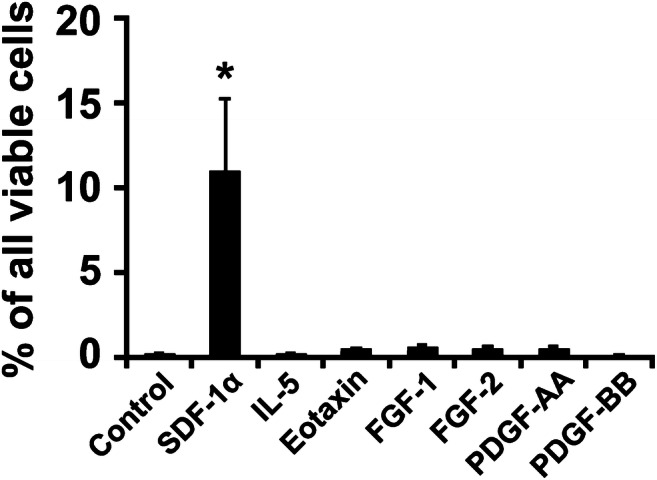
The effects of various cytokines on migration of neoplastic eosinophils. The eosinophil cell line EOL-1 carrying *FIP1L1-PDGFRA* was loaded in the upper chambers of a Boyden-type double-chamber system. The lower chambers were supplemented with control medium or medium containing recombinant human SDF-1ɑ (25 ng/ml), IL-5 (100 ng/ml), eotaxin (500 ng/ml), FGF-1 (100 ng/ml), FGF-2 (100 ng/ml), PDGF-AA (100 ng/ml), or PDGF-BB (100 ng/ml). After 4 h (5% CO_2_, 37 °C), the numbers of viable migrated cells collected in the lower chambers were measured by flow cytometry. Results are expressed as percent of all viable cells (100% input) and represent the mean ± S.D. of 3 independent experiments. Asterisk (*), *p* < 0.05 compared to medium control. Abbreviations: SDF-1, stroma cell–derived factor; IL-5, interleukin-5; FGF, fibroblast growth factor; PDGF, platelet-derived growth factor

Eosinophils derive from pluripotent and lineage-related hematopoietic precursor cells [4, 20–25]. Bi-lineage-restricted progenitor cells giving rise to eosinophils and basophils (CFU-eo/ba) are commonly detected in the BM and peripheral blood of healthy individuals, patients with reactive (inflammatory) disorders, and those with myeloproliferative neoplasms [20–25]. The development of eosinophils from their multipotent and lineage-specific precursor cells is coordinated by a network of transcription factors, growth-promoting cytokines, and growth-inhibitory mediators. Growth factors for eosinophil precursor cells include interleukin (IL)-5, granulocyte/macrophage colony-stimulating factor (GM-CSF), and IL-3 (Table 2) [26–28]. These growth-modulating cytokines are primarily synthesized and secreted by (activated) T cells, mast cells, and stromal cells. Receptors for these cytokines are expressed on multipotent myeloid precursor cells, eosinophil-committed progenitors, immature eosinophils (all morphological stages), and mature eosinophils [29–31]. Correspondingly, these growth factors mediate not only the proliferation of eosinophil precursor cells but also migration, adhesion, survival, and activation of mature eosinophils (Table 2) [32–34]. Apart from the classical eosinophilic growth regulators, other cytokines, such as IL-13, platelet-derived growth factor (PDGF), or nerve growth factor (NGF), may also contribute to the differentiation and maturation of normal and neoplastic eosinophils (Table 2) [35–37]. In addition, a number of chemokines, such as stroma cell–derived factor-1 (SDF-1 = CXCL12), CCL5 (RANTES), CCL11 (eotaxin-1), CCL24 (eotaxin-2), CCL26 (eotaxin-3), or platelet-activating factor (PAF), can induce eosinophil migration, activation, and/or chemotaxis (Table 2) [38–48]. The most potent chemotactic molecule for neoplastic eosinophils may be SDF-1 (Fig. 1) [48].

**Table 2:** Stimuli and their receptors that alter various human eosinophil functions

Stimulus	The effects on eosinophils and/or their precursor cells	Receptor/s (R)
Differentiation-inducing		
IL-3	Differentiation, survival, adhesion, migration, activation, priming	IL-3R = CD123+CD131
IL-5	Differentiation, survival, adhesion migration, activation, priming	IL-5R = CD125+CD131
GM-CSF	Differentiation, survival, adhesion, migration, activation, priming	GM-CSFR = CD116+CD131
Growth- or survival-promoting		
PDGF	Survival*, activation?	PDGFRA/B
FGF	Survival*, activation?	FGFR1
IL-25	Survival, activation	IL-25R
IL-27	Survival, activation	IL-27R
Inhibitory		
TGFß1	Inhibitory (growth, activation)	TGFß1R
TGFß2	Inhibitory (growth, activation)	TGFß2R
IFN-alpha	Inhibitory (growth)	IFN-alpha-R
IFN-gamma	Inhibitory (growth, migration)	IFN-gamma-R
IL-10	Inhibitory (activation, survival)	IL-10R
IL-12	Inhibitory (activation)	IL-12R
Activating and/or migration-inducing		
C3a, C5a	Chemotaxis, activation	C3aR, C5aR
PAF	Chemotaxis, activation	PAF-R
SDF-1 (CXCL12)	Chemotaxis	CXCR4
RANTES (CCL5)	Chemotaxis, activation	CCR3
MCP-3 (CCL7)	Chemotaxis, activation	CCR3
MCP-4 (CCL13)	Chemotaxis, activation	CCR3
Eotaxin (CCL11)	Chemotaxis, activation	CCR3
Eotaxin-2 (CCL24)	Chemotaxis, activation	CCR3
Eotaxin-3 (CCL26)	Chemotaxis, activation	CCR3
IL-2	Activation, PRIMING	IL-2RA/CD25
IL-4	Priming for chemotaxins	IL-4R/CD124
IL-13	Activation?	IL-13R
IL-16	Activation, priming	CD4,CD9(?),CCR3
IL-33	Activation, adhesion, migration	IL-33R/ST2
VEGF	Chemotaxis, activation	VEGFR-1/FLT-1
Angiopoietin-1	Chemotaxis, activation?	Tie-2/TEK

^*^In hematologic malignancies with HE where oncogenic mutant forms of *PDGFR* or *FGFR* are expressed by neoplastic (progenitor) cells, the differentiation of eosinophils is considered to be triggered primarily by these oncogenic mutant forms of PDGFR/FGFR

*PDGF* platelet-derived growth factor, *FGF* fibroblast growth factor, *PAF* platelet-activating factor, *IL* interleukin, *GM-CSF* granulocyte/macrophage colony-stimulating factor, *TGF* transforming growth factor, *IFN* interferon, *CCL* chemokine ligand, *CCR* chemokine receptor, *MCP* monocyte chemotactic protein, *VEGF* vascular endothelial growth factor

The development, survival, and function of eosinophils are also controlled by diverse inhibitory cytokines, their receptors, and other inhibitory receptors and “negative-regulators” [49–55]. Among cytokines, transforming growth factor-beta (TGF-beta), interferon-alpha (IFN-alpha), and IFN-gamma have been described to inhibit cytokine-induced differentiation of human eosinophils from their progenitor cells (Table 2) [51–54]. Moreover, certain cytokines, like IFN-gamma, can block cytokine-mediated migration of eosinophils [56]. All these cytokine-induced effects on eosinophils and their precursor cells are considered to be mediated via specific cell surface receptors. Eosinophils also display receptors for glucocorticosteroids which inhibit growth, activation, and survival of these cells [57, 58].

## Phenotype and target expression profile of eosinophils in health and disease

Eosinophils express a unique composition of cell surface receptors relevant to adhesion, homing, and migration in tissues [4, 5, 59–69]. Some of these receptors contribute to the transmigration of eosinophils across endothelial monolayers and thus to the homing of eosinophils and their precursor cells in tissues, which is critical for the development of tissue HE found in patients with HE-related organ damage. Eosinophils display the C3bi receptor (CD11b/CD18), leukocyte function antigen-1 (LFA-1 = CD11a/CD18), L-selectin, E- and P-selectin ligands, low levels of sialyl Lewis x, and intercellular adhesion molecules (ICAM) [59–69]. Moreover, eosinophils display leukosialin (CD43) and the leukocyte-invasion receptor CD44. Whereas selectins and their receptors are considered to mediate eosinophil rolling and tethering on vascular cells, integrins and other receptors cause firm binding of eosinophils to endothelial cells prior to transmigration into tissues. Various cytokines and peptides may promote the expression and/or function of adhesion antigens on eosinophils. By contrast, glucocorticosteroids and some of the anti-inflammatory cytokines may block expression of adhesion receptors on eosinophils and thus adhesion of eosinophils and their transmigration into tissues [70, 71]. On the other hand, glucocorticosteroids may even upregulate expression of certain homing receptors, like CXCR4 (receptor for SDF-1), on eosinophils [72].

Eosinophils also have on their surface several biologically relevant, activation-linked cell surface membrane antigens, including complement receptors, toll-like receptors, Fc receptors, gangliosides, glycoproteins, and Siglec molecules, such as Siglec-8 [49, 73–78]. In addition, activated eosinophils and neoplastic eosinophils can display CD25 (Table 2) [79]. Among all these receptors, Siglec-8 appears to be a rather specific surface molecule that is expressed on eosinophils and their progenitor cells but not to any significant degree on other blood leukocytes [49, 78].

**Fig. 2:**
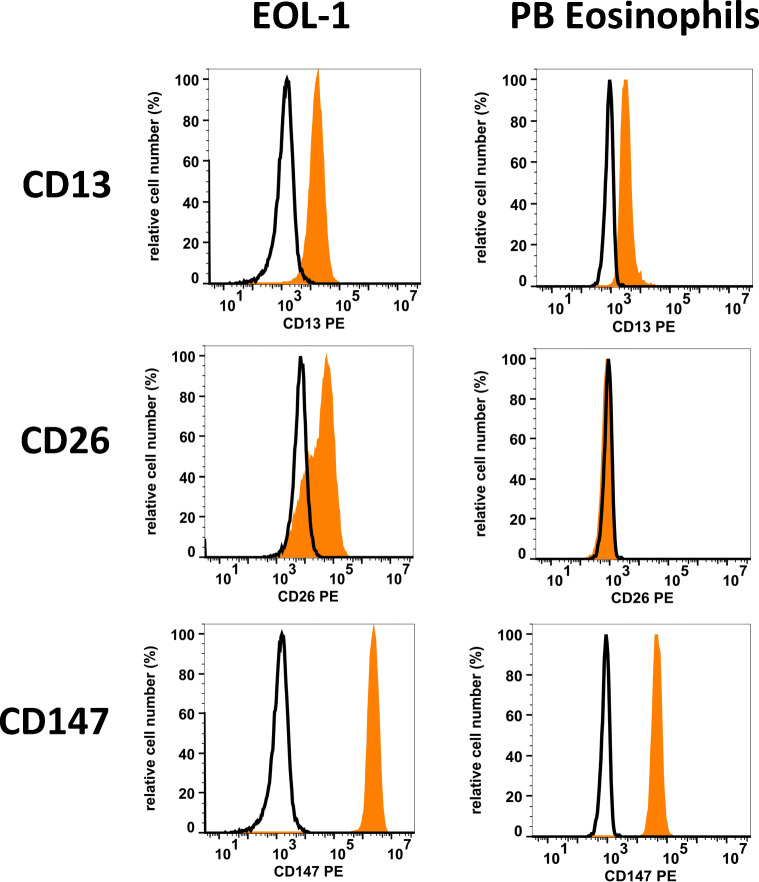
Expression of corona virus receptors on human eosinophils. EOL-1 cells (left panels) and normal peripheral blood (PB) eosinophils were stained with PE-conjugated antibodies against three corona virus receptors, namely CD13 (aminopeptidase-N: clone WM15), CD26 (DPPIV: clone M-A261), and CD147 (basigin: clone HIM-6). Antibody reactivity (orange histograms) was analyzed by flow cytometry. The isotype-matched control antibody is also shown (black open histograms). Abbreviations: CD, cluster of differentiation; PE, phycoerythrin; DPPIV, dipeptidyl-peptidase IV

Finally, eosinophils display a number of cell surface receptors for certain viruses and related antigens, including the corona virus receptors CD13 and CD147, the measles virus receptor CD46, and the Echo-/Coxackie virus receptor CD55 (DAF) (Table 3 and Fig. 2) [59]. Once activated by cytokines, such as IFN-gamma or tumor necrosis factor alpha (TNF-alpha), eosinophils may display additional virus receptors, such as the rhinovirus receptor CD54 (ICAM-1) [80]. Whether eosinophils can serve as a reservoir for certain viruses or as effector cells of tissue damage following virus infection, such as SARS-CoV-2 infection, remains unknown. It is worth noting in this regard that tissue inflammation and tissue damage (lung) induced by certain corona viruses are sometimes resembling features of HES. On the other hand, however, HE is usually not seen in patients with SARS-CoV-2 infections.

**Table 3:** Cell surface receptors for viruses expressed on human eosinophils and EOL-1 cells

			Expressed on	
Antigen-receptor	CD	Virus	Eosinophils	EOL-1
Corona virus receptors				
Aminopeptidase-N	13	Corona virus	+	+
DPPIV	26	Corona virus	−	+
Basigin	147	Corona virus	+	+
Other virus receptors				
T4 antigen	04	HIV	−	−
Complement R2 (CR2)	21	EBV	−	−
Membrane co-factor protein	46	Measles virus	+	+
VLA-2	49b	Echo virus	−	−
VLA-3	49c	Herpes virus-8	−	−
VLA-6	49f	Papilloma viruses?	+	+
ICAM-1	54	Rhino virus	+/−*	+
Decay accelerating factor	55	Echo virus-70	+	+/−
MACIF	59	African swine virus	+	+
Tetraspan-28	81	HCV	+	+
Nectin-1 (PVRL1)	111	Env gD of herpes virus	+/−	+/−
Nectin-2 (PVRL2)	112	Env gD of herpes virus	+	+
PVR	155	Polio virus	+/−	+
CXCR4	184	HIV	+	+
CCR5	195	HIV	−	n.t.
JAM-A	321	Reo virus	+	+

Results refer to data published in the literature or data obtained in our laboratories by flow cytometry. In these experiments, expression of virus receptors on Siglec-8-positive blood eosinophils and the eosinophilic cell line EOL-1 was examined by monoclonal antibodies and multi-color flow cytometry

^*^Cytokine-activated eosinophils may express the cell surface antigen ICAM-1 (CD54)

*DPPIV* dipeptidyl-peptidase IV, *HIV* human immunodeficiency virus, *EBV* Epstein Barr virus, *VLA* very late antigen, *ICAM* intercellular adhesion molecule, *HCV* hepatitis C virus, *n.t.* not tested

## Eosinophil-derived molecules and their impact in HE-induced organ damage

Eosinophils produce an array of proinflammatory mediators and cytokines, including interleukins, chemokines, and other compounds [1–5, 81–85]. Several of these molecules, such as the chemokines, can recruit and/or activate leukocytes in affected organ sites. Other molecules contribute to the mobilization and activation of local microenvironmental cells, such as fibroblasts or endothelial cells, and thereby trigger tissue fibrosis, angiogenesis, and tissue remodeling [81–85]. In addition, eosinophils generate lipid-based mediators, including cysteinyl leukotrienes and prostaglandins which contribute to tissue inflammation and organ dysfunction (Table 4) [81–85].

**Table 4:** Eosinophil products and their potential impact in the etiology of HE and HES

Eosinophil product	Effects potentially relevant to HE-related organ damage = HES*
Cytokines/interleukins	
GM-CSF	Leukocyte/eosinophil activation
IL-1	Endothelial cell activation, inflammation
IL-2	Activation of T lymphocytes
IL-3	Eosinophil accumulation and activation
IL-4	B cell maturation and mast cell development
IL-5	Eosinophil accumulation and activation
IL-6	Eosinophil accumulation and activation
IL-8	Leukocyte recruitment/activation
IL-13	Bronchial hyperreactivity, mucus production, B cell maturation
TGF-alpha	Fibrosis, growth inhibition
TGF-beta	Fibrosis, growth inhibition
TNF-alpha	Endothelial activation, inflammation, cachexia
OSM	Fibrosis, angiogenesis**
Chemokine ligands	
Eotaxin (CCL11)	Further eosinophil recruitment
MIP-1-alpha (CCL3)	Leukocyte recruitment & activation
RANTES (CCL5)	Leukocyte recruitment & activation
Eosinophil-derived basic proteins	
Eosinophil cationic protein (ECP)	Direct toxic effects, mucus secretion, fibrosis
Eosinophil-derived neurotoxin (EDN)	Direct toxic effects, TLR2 ligand effects, RNase
Eosinophil peroxidase (EPO)	Direct toxic effects, leukocyte activation
Major basic protein (MBP)	Direct toxic effects, leukocyte activation
Toxic and immunoregulatory enzymes	
Acid phosphatase	Direct toxic effect
Arylsulphatase B	Lysosomal hydrolase
Catalase	Direct toxic effects
Hexosaminidase	Direct toxic effects
Histaminase	Histamine degradation
Lysophospholipase	Direct toxic effects
Nonspecific esterases	Direct toxic effects
Phospholipase D	LFA-dependent adhesion
Membrane-derived lipid-compounds	
LTC4	Mucus secretion
PAF	Bronchoconstriction, edema formation
PGE1 & PGE2	Diverse effects on platelets, endothelial cells, fibroblasts and other tissue cells
15–HETE	Diverse effects on blood and tissue cells
TXB2	Platelet aggregation
HE-Related DNA Traps	Direct toxic effect & prothrombotic effects
Fibrinolysis blocker	
PAI-2	Anti-fibrinolytic and prothrombotic effects

^*^The direct toxic effects of the eosinophil-derived mediators, proteins and enzymes are often directed against certain microbes such as bacteria (antimicrobial effects) but may also be directed against various physiologic cells, especially when the number of eosinophils and the concentrations of these eosinophil-derived compounds increase in tissues, which may lead to tissue damage and thereby HES

^**^Neoplastic eosinophils triggered by various PDGFR mutant forms express and release increased amounts of OSM, certain chemokines, and other microenvironmental cell regulators compared to normal blood eosinophils

*HE* hypereosinophilia, *IL* Interleukin, *GM-CSF* granulocyte/macrophage colony-stimulating factor, *TGF* transforming growth factor, *TNF* tumor necrosis factor, *OSM* oncostatin M, *PAF* platelet-activating factor, *PGE* prostaglandin E, *TX* thromboxane, *PAI-2* plasminogen activator inhibitor-2

Eosinophils can synthesize and release a number of specific cytotoxic effector proteins, including eosinophil cationic protein (ECP), eosinophil major basic proteins (MBP1 and MBP2), eosinophil peroxidase (EPO), and eosinophil-derived neurotoxin (EDN) (Table 4) [86, 87]. These compounds and other molecules, such as the eosinophil-derived extracellular DNA traps, contribute to microbe killing and thus innate immune host defense [86–88]. These DNA traps produced by eosinophils may also contribute to thrombophilia that is often associated with HE. Eosinophils also produce and release Charcot-Leyden crystals (CLC) which consist of the eosinophil granule protein Galectin-10 and may contribute to type 2 immunity and tissue inflammation [89]. Finally, eosinophils produce a number of repair molecules that may be involved in tissue homeostasis after an inflammatory or toxic reaction in local tissue sites. Likewise, eosinophil-derived molecules may counteract or degrade vasoactive molecules such as histamine (eosinophil-derived histaminase).

Concerning HE-related organ damage, little is known about the exact role each of the eosinophil-derived substances play in various disease contexts. It is generally appreciated that eosinophil-derived chemokines, cytokines, and mediators contribute to leukocyte recruitment, tissue inflammation, thrombosis, fibrosis, and subsequent tissue damage (Table 4). Eosinophil products known to mediate fibrosis and thrombosis by affecting endothelial cell and/or platelet function include among others, DNA traps, plasminogen activator inhibitors, and toxic proteins [87–94].

These eosinophil-derived mediators and cytokines may all act together to cause thrombophilia, tissue fibrosis, and thus organ dysfunction or even tissue damage in patients with HE/HES in various contexts and pathologies.

## Definition and classification of HE and HES

Eosinophilia can be divided into relative and absolute eosinophilia, slowly progressing versus suddenly occurring eosinophilia, transient versus persistent eosinophilia, and mild (up to 1500/μL) versus marked eosinophilia (>1500/μL). When eosinophilia is marked and persists for several months, the term blood HE is appropriate [7–11, 95]. Blood HE may or may not be accompanied by tissue HE in various organ systems. The term tissue HE is less well defined than the term blood HE. In general, tissue HE is characterized by a marked increase in eosinophils and/or a substantial deposition of eosinophil-derived proteins, like MBP, in tissue sections in affected organs [6–11, 91, 96, 97]. In patients with chronic tissue HE, eosinophils usually undergo cell death and disappear in local sites after activation and release of their proteins, so that the predominant histological finding of tissue HE may be deposition of MBP and other eosinophil-derived proteins without eosinophil accumulations [91, 96].

Based on the underlying etiology, several variants of HE have been defined [10, 11]: a very rare hereditary (familial) variant (HE_F_), HE of unknown significance (HE_US_) where the cause and clinical impact of HE remain unknown, primary (neoplastic) HE (HE_N_) where clonal (neoplastic) eosinophils are detected, and secondary, reactive HE (HE_R_) where normal (activated, non-neoplastic) eosinophils expand in a reactive (often inflammatory) process (Table 5) [10, 11]. In rare cases, HE_R_ is induced by a neoplastic process such as a lymphoma, a gastrointestinal tumor, or a lung carcinoma. In most patients with HE_R_, HE may be induced by eosinotropic cytokines, like IL-5.

**Table 5:** Classification of hypereosinophilia (HE)

Variant of HE	Abbreviation	Features
Hereditary (familial) HE	HE_FA_	Familial clustering, no evidence of a hereditary immunodeficiency, no evidence of a reactive or neoplastic underlying disease, and no signs or symptoms indicative of HES
HE of unknown significance	HE_US_	No known underlying etiology of HE, no positive family history, no evidence of a reactive or neoplastic condition or disorder underlying HE
Secondary (reactive) HE	HE_R_	Underlying reactive condition or disease that explains HE, no evidence for a clonal bone marrow disease that explains HE*
Primary (clonal/neoplastic) HE	HE_N_	Underlying stem cell, myeloid, or eosinophil neoplasm inducing HE*

^*^In primary, neoplastic HE (HE_N_), eosinophils are considered to be clonal cells derived from neoplastic stem cells, whereas in reactive HE (HE_R_), eosinophils are considered to be reactive (non-clonal) cells triggered by eosinophiliopoietic cytokines such as interleukin-5

*HE* hypereosinophilia, *HES* hypereosinophilic syndrome

Patients with unexplained HE may be asymptomatic for several months or even years. As mentioned, these patients are classified as HE_US_ (Table 5) [10, 11]. In these patients, no underlying (neoplastic or non-neoplastic) disease and no HE-induced organ damage are detected. Several of these patients may develop signs and symptoms of eosinophil-mediated organ damage during follow-up. When HE leads to organ damage in such patients, the final diagnosis is “hypereosinophilic syndrome” (HES) [10, 11].

Like HE, HES is also divided into distinct variants based on underlying etiologies: idiopathic HES (unknown etiology: HES_US_), primary (neoplastic) HES with an underlying clonal myeloid or stem cell disorder (HES_N_), and secondary (reactive) HES in which a related non-neoplastic or paraneoplastic condition is detected and is responsible for the expansion of (activated) eosinophils (HES_R_) (Table 6) [10, 11].

**Table 6:** Classification of hypereosinophilic syndromes (HES) and related disorders

Variant	Typical features
Idiopathic HES	No underlying cause of HE, no evidence of a reactive or neoplastic condition/disorder underlying HE, and end organ damage attributable to HE
Primary (neoplastic ) HES (HES_N_)	Underlying stem cell, myeloid, or eosinophil neoplasm classified according to WHO and end organ damage attributable to HE. Eosinophils are considered (or shown) to be neoplastic (clonal) cells*; HES_N_
Secondary (reactive) HES(HES_R_)	Underlying condition/disease where eosinophils are considered non-clonal cells, and HE is considered to be cytokine-driven (HES_R_), and end organ damage attributable to HE
Special variants/syndromes	
Lymphoid variant of HES (HES-L)**	Abnormal clonal T cells often detected, HES-related organ damage found, but angioedema and elevated IgM usually absent
Episodic angioedema and eosinophilia (Gleich’s syndrome)	Abnormal clonal T cells usually detected, angioedema, and elevated polyclonal IgM
Eosinophilic granulomatosis with polyangiitis = (EGPA) = Churg-Strauss syndrome	Polyangiitis, necrotizing angiitis, asthma, lung infiltrates; in a subset of patients, ANCA can sometimes be detected (ANCA^+^ form of EGPA)
Eosinophilia myalgia syndrome (EMS)	Myalgia, muscle weakness, cramping, skin rash, dyspnea, fatigue
Omenn syndrome	Autosomal recessive disease with hypomorphic missense mutations in immunologically relevant genes, like *RAG1* or *RAG2*, severe combined immunodeficiency with recurrent infections, auto-reactive T cells, skin rash, erythroderma, splenomegaly, lymphadenopathy, GvHD-like symptomatology
Hyper-IgE syndrome	Hereditary immunodeficiency syndrome, elevated serum IgE, recurrent bacterial infections, often with eczema and facial anomalies. Autosomal dominant variant: *STAT3* mutations. Autosomal recessive variant: *DOCK8* mutations

^*^Clonality of eosinophils is often difficult to demonstrate or is not examined. However, if a myeloid or stem cell neoplasm known to present typically with clonal HE, is present, or a typical molecular defect is demonstrable (e.g., *PDGFR* or *FGFR* mutations or *BCR-ABL1*), eosinophilia should be considered clonal

^**^The lymphoid variant of HES is regarded a special form of secondary HES, although its exact nature and pathogenesis remain controversial

*HE* hypereosinophilia, *HES* hypereosinophilic syndrome, *ANCA* anti-neutrophil cytoplasmic antibodies

A number of special variants of HES have also been described. One is the so-called lymphoid variant of HES (HES-L) [98–101]. Although no solid diagnostic criteria are available, HES-L exhibits several features of (and may therefore be regarded as) reactive HES (HES_R_). Typically, T lymphocytes in these patients exhibit an abnormal phenotype (such as CD3^−^/CD4^+^). In most cases, a clonal T cell receptor rearrangement is found. The clinical course of patients with HES-L is often indolent without signs of progression. However, some of the patients may progress to an overt T cell lymphoma.

A number of organ systems may be affected in HES, including the heart, lung, skin, gastrointestinal tract, and the central nervous system [6–11, 102–104]. A particularly devastating manifestation of HES is the thromboembolic state which may include stroke, intracavitary thrombi in the heart, and vascular (arterial and/or venous) thrombosis. In addition, endomyocardial fibrosis, chronic tissue inflammation, and ulcerations in the skin are often seen in patients with HES [6, 10, 11, 102–104]. Such HES-associated organ damage may develop in all variants of HES, independent of the underlying etiology.

Endomyocardial fibrosis and thrombus formation as well as cardiac arrest are also seen in patients with eosinophil-rich neoplasms carrying the *FIP1L1-PDGFRA* fusion gene or other *PDGFR* variants. Most of these patients respond well to imatinib and early treatment with this drug may prevent irreversible organ damage in these patients [105–108]. Therefore, it is of considerable importance to perform diagnostic investigations, including molecular studies, as early as possible and to start treatment with imatinib before irreversible organ damage develops.

## Diagnostic evaluations, staging, and diagnostic algorithms

Initial investigations in patients with HE include a detailed case history, especially travel histories, food, and toxin exposures, and certain infections and infestations. In the case of a suspected helminth infection, stool examinations and serology tests should be performed [8–11, 13]. In addition, bacterial and viral infections must be excluded or diagnosed by appropriate serology and molecular assessments [8–11, 13].

When no causative infection, drug, or toxin can be identified, the patient is examined for the presence of allergic (atopic) diseases, chronic inflammatory diseases, blood cell disorders, and other neoplastic conditions [8–19, 109, 110]. It is also worth noting that HE may be caused by more than one trigger or underlying disease. Therefore, diagnostic evaluations in HE should always encompass all major etiologies.

In all patients with HE, detailed laboratory examinations are performed, including blood counts with differential counts, serum chemistry including a basal serum tryptase level, inflammation markers (including fibrinogen and CRP), autoantibodies, serum IgE, and vitamin B12 [10, 11, 109, 110]. In those with a suspected hematologic disease, molecular screen parameters are applied, including assays detecting *PDGFR*-related fusion gene products, such as *FIP1L1-PDGFRA*, *KIT* D816V, *JAK2* V617F, *BCR-ABL1*, and clonal T cell receptor rearrangement [10, 11, 13–18]. When these parameters disclose negative results, next generation sequencing (NGS) and BM studies should be considered. BM investigations are also performed when these screens show a positive test-result or other signs and symptoms suggest that the patient is suffering from a hematopoietic neoplasm. In patients with suspected eosinophilic leukemia or other myeloid neoplasms, the underlying disease is diagnosed based on WHO criteria [10, 11, 13–19]. Investigations in these patients include morphologic studies of eosinophils and other cell types on good-quality BM and blood smears (stained with Wright-Giemsa) and a BM core biopsy with histology and immunohistochemistry to define the number, distribution, and phenotype of myeloid (precursor) cells, mast cells, megakaryocytes, and other BM cells, and to document or exclude BM fibrosis and myelodysplasia. Additional examinations include a detailed flow cytometry analyses, conventional cytogenetics, and fluorescence in situ hybridization (FISH) and molecular studies, including PCR and NGS [10, 11, 16–18]. PCR and FISH are required to detect certain fusion gene variants, such as *FIP1L1-PDGFRA* and the related *CHIC2* deletion (by FISH) [10, 11, 16–18]. In patients with suspected lymphoid HES, a detailed flow cytometric analysis of lymphocytes should be performed, with the aim to exclude or identify aberrant populations, such as CD3^−^/CD4^+^ T cells which are often detected in the lymphoid variant of HES = HES-L [8–11, 98–101].

In all patients with suspected HES, HE-induced organ damage should be documented by appropriate staging investigations, including physical examination with a detailed inspection of the skin, cardiologic assessments, measurements of serum troponins and pro B-type natriuretic peptide (proPNB), an electrocardiogram and echocardiogram, assessment of pulmonary function, chest X-ray, abdominal imaging, computed tomography, cardiac MRT and biopsy, and gastrointestinal examinations with endoscopic biopsy studies [6–13, 109, 110].

## Underlying pathologic conditions and differential diagnoses

Once HE has been diagnosed, its etiology must be determined and the question as to whether (or not) the patient is suffering from HES (HE-induced organ damage) has to be clarified. In most patients with HE, an underlying reactive condition or neoplastic disease will be diagnosed, leading to the provisional diagnosis HE_R_ [10, 11]. In rare cases, a familiar variant of HE is identified (Table 5). When no underlying disease, no positive family history, no organ damage, and no other related disease or syndrome associated with HE is detected, the provisional diagnosis HE_US_ is appropriate [11].

A number of reactive conditions and underlying diseases can produce HE (Table 1). Specifically, reactive HE (HE_R_) may develop not only in patients with an underlying parasitic, bacterial, fungal, or viral infection but also in patients with IgE-dependent or IgE-independent allergies and patients with chronic inflammatory (auto-immune-mediated) disorders. In a subset of these patients, a reactive HES (=HES_R_) is diagnosed. In addition, HE_R_ may be identified in cancer patients, for example in adenocarcinomas developing in the lung, cervix, or the gastrointestinal (GI). There are also a few hematological malignancies where eosinophilia is typically reactive in nature, such as in Hodgkin’s lymphoma, T cell lymphomas, or B cell acute lymphoblastic leukemia.

Myeloid neoplasms that typically present with clonal/neoplastic HE (HE_N_) include Ph^+^ CML, certain variants of AML, and advanced SM (Table 1) [10, 11, 14–19]. Moreover, marked eosinophilia (and sometimes HE) may develop in MDS, MPN, and MDS/MPN overlap neoplasms, including chronic myelomonocytic leukemia (CMML). These neoplasms should be diagnosed and classified using WHO criteria [10, 11, 14–19]. Based on these criteria, hematopoietic neoplasms producing eosinophilia are initially classified according to the presence or absence of certain gene variants, including abnormalities (mutant forms) of *PDGFRA*, *PDGFRB*, or *FGFR1*, or the *PCM1-JAK2* fusion gene. When one of these genetic lesions is detected, the final histopathological and hematological diagnoses still need to be determined, since the underlying disease may be an acute leukemia, an eosinophil-rich chronic leukemia, a MPN, or a CMML [10, 11, 14–19]. Additional gene variants that may be detected in patients with myeloid neoplasms and eosinophilia include, among others, *ETV6-ABL1*, *STAT5B* N642H, and *JAK2*ex13InDel. Both the type of molecular lesion and the type of underlying disease have prognostic and therapeutic implications. For example, most (chronic) hematopoietic neoplasms exhibiting the FIP1L1-PDGFRA fusion protein are responsive to treatment with imatinib [105–108] whereas this is not the case in patients with a *FGFR1*-mutated malignancy. Indeed the prognosis changes when the *FIP1L1-PDGFRA*+ neoplasm turns out to be an acute leukemia.

Another important aspect is lineage involvement. For instance, in patients with *PDGFR* variants, lymphoid involvement is rarely seen, while patients with *FGFR1*-mutated neoplasms with concomitant HE may present with a stem cell malignancy exhibiting lymphoid and myeloid involvement. The WHO classification also includes the category “chronic eosinophilic leukemia — not otherwise specified” (CEL NOS) [14–18]. However, there are patients who present with a more acute form of an eosinophil leukemia. Therefore, the international cooperative study group on eosinophil disorders (ICOG-EO) proposed to delineate between acute eosinophilic leukemia (AEL) and chronic eosinophilic leukemia (CEL), based on the percentage of blast cells [11].

In patients with HE in whom eosinophils are ≥30% and blast cells are ≥20% of all nucleated (BM or blood) leukocytes, the diagnosis is AEL [11]. In patients with HE in whom eosinophils are ≥30% and blast cells are <20% of all nucleated (BM or blood) leukocytes, the diagnosis is CEL [11].

Finally, there are a number of organ-specific disorders and systemic syndromes defined by HE and HES or HES-like pathologies. Although these syndromes have been separated from the classical variants of HES, the clinical presentations and courses often resemble HES [11]. Organ-specific disorders include eosinophil inflammatory states, such as eosinophilic esophagitis, eosinophilic gastritis, eosinophil duodenitis, eosinophil colitis, eosinophilic pneumonia, and eosinophilic hepatitis. In most of these reactive conditions, the disease is triggered by certain eosinopoietic cytokines such as IL-3, IL-5, IL-13, or GM-CSF, and at least for eosinophilic esophagitis/gastritis, ingestion of certain foods can be a crucial trigger. Systemic (multi-organ) syndromes include, among others, episodic angioedema and eosinophilia (Gleich’s syndrome), eosinophilic granulomatosis with polyangiitis (EGPA; formerly Churg-Strauss syndrome = CSS), eosinophilia myalgia syndrome (EMS), Omenn syndrome, and the Hyper-IgE syndrome.

A detailed description of all these pathologies and syndromes is beyond the scope of this article. We refer the reader to the available literature. Gleich’s syndrome is defined by recurrent angioedema, peripheral blood HE, and elevated polyclonal IgM [111–113]. In several of these patients, phenotypically abnormal (activated) T cells (CD4^+^ T cells with decreased CD3 expression) can be documented [101, 113]. Based on this notion, the Gleich’s syndrome is also regarded as special form or manifestation of HES-L. Typical features of EGPA/CSS are asthma, a necrotizing vasculitis, and eosinophilia [114–116]. In a subset of these patients, anti-neutrophil cytoplasmic antibodies (ANCA) are detected [114–116]. EMS is characterized by myalgia, neurologic symptoms, and skin exanthema. Epidemic cases of EMS have been reported. In these patients, exposure to L-tryptophan has been described [117–119]. Therefore, the condition has also been termed toxic oil syndrome [117–119]. Both Omenn syndrome and the Hyper-IgE syndrome are rare inherited immunodeficiency disorders accompanied by eosinophilia.

## Contemporary management of patients with HE-related disorders

For patients with HE_US_ and HE_F_ with a stable (silent) clinical course, a wait-and-watch strategy may be chosen, provided that no signs or symptoms indicative of organ dysfunction or organ damage develop [10, 11]. In fact, both HE_US_ and HE_F_ are provisional diagnoses and in both instances, a hematologic disease or reactive disease with or without organ damage may develop in the follow-up [8–14].

The reactive form of HE (HE_R_) is best managed by treating and (if possible) eradicating the underlying disease or pathology [8–11, 105–109, 120–124]. When eradication is not possible, symptomatic therapy may be sufficient to control problems related to eosinophil activation and HE in these patients. In many of these patients, organ damage (=HES_R_) can be prevented by administration of glucocorticosteroids and/or other anti-inflammatory drugs [8–11, 109, 120–124]. In patients with severe eosinophilic granulomatosis and polyangiitis (EGPA/CSS), additional drugs, such as cyclophosphamide, may be required. It is worth noting that corticosteroids can induce apoptosis in eosinophils and their precursor cells and also suppress synthesis of eosinophil-activating cytokines and chemokines in T lymphocytes and other cells. In patients with idiopathic HES and HES-L, glucocorticoids are also recommended. However, side effects of long-term glucocorticoid therapy may be a clinical challenge and may be dose-limiting. Glucocorticosteroid-sparing agents may help in these cases. Apart from conventional drugs, including hydroxyurea and IFN-alpha, the anti-IL-5 antibody mepolizumab has been shown to be a safe and effective corticosteroid-sparing agent in these patients and is the only biologic approved for the treatment of HES in the USA [125–129].

Most patients with primary (neoplastic) HE and HES are not responding to corticosteroids or other anti-inflammatory agents. In these patients, specific targeted drugs and/or (more) intensive therapies are required to bring eosinophil counts and HE under control [10, 11, 16–18, 107, 122–124, 128–132]. The classes and type of drugs are selected based on the underlying neoplasm, the molecular drivers detected, and the overall situation in each case. Therefore, it is essential to define the molecular defects and the target expression profiles in clonal cells and to establish the exact histomorphological diagnosis and the extent of organ involvement and organ damage in each case [8–19].

In many patients with eosinophil-rich neoplasms, a mutation in *PDGFRA* or *PDGFRB* is detected [10, 11, 14–18]. The most common genetic variant identified in such patients is *FIP1L1-PDGFRA*. The respective fusion gene product, FIP1L1-PDGFRA, is a well-established target of imatinib and other PDGFR-directed TKI. Several other fusion gene targets of imatinib have also been described in eosinophil-rich BM neoplasms. Most fusion gene products involving PDGFRA or PDGFRB receptors are sensitive to imatinib, whereas oncogenic FGFR1 mutants are resistant [11, 16–18, 107, 128, 130–132].

In patients with chronic, eosinophil-rich, neoplasms exhibiting FIP1L1-PDGFRA or other imatinib-sensitive PDGFR variants, imatinib is regarded standard first-line therapy [10, 11, 16–18, 105–109, 130–132]. Although most patients show a long-lasting response to 100 mg imatinib daily, a few patients require a dose of 200 or 400 mg per day. A very few patients develop resistant disease, often in the context of rare secondary mutations in *FIP1L1-PDGFRA* [107, 130–134]. For these patients and for imatinib-intolerant cases, other PDGFR-targeting drugs, such as sorafenib or midostaurin, should be considered provided that the disease still presents as a chronic PDGFR-dependent neoplasm. Alternatively, these patients are treated with hydoxyurea or more intensive therapy, and the same holds true for patients in whom the disease progresses into an acute resistant leukemia. In some of these resistant patients, intensive chemotherapy and stem cell transplantation have to be considered. Patients with an advanced *JAK2*-mutated disease, including cases with *JAK2* V617F and those with *PCM1-JAK2*, JAK-targeting drugs, such as ruxolitinib or fedratinib, may be considered. In patients with *FGFR1*-mutated chronic neoplasms with HE, treatment options are limited. In some of these patients, transient responses to midostaurin (in a *ZNF198-FGFR1*+ disease) or ponatinib (in BCR-FGFR1+ leukemia) have been described. However, in most patients, no long-lasting effects are seen. For patients with *FGFR*-mutated neoplasms, several FGFR-targeting drugs, such as pemigatinib, have recently been developed and are currently tested in clinical trials. However, most of these patients have drug-resistant disease and progress rapidly, often in form of an aggressive stem cell disease or a leukemia/lymphoma syndrome (8p11 syndrome). For these patients, intensive poly-chemotherapy and allogeneic stem cell transplantation combined with specific targeted drugs may be a reasonable approach to consider. However, even such intensive therapies may not always lead to long-term disease control or cure in patients with the 8p11 syndrome.

## Concluding remarks and future perspectives

Eosinophils are multifunctional effector cells of the immune system that are involved in host defense, tissue remodeling, and tissue repair. Once activated, eosinophils may release toxic substances that support host defense and may also cause tissue damage, especially in hypereosinophilic states. HE may develop in the context of various hematologic neoplasms and in certain reactive states. In all these patients, it is important to (i) document or exclude a related neoplastic or non-neoplastic disease, and to (ii) document or exclude the presence of HE-related organ damage which leads to the diagnosis of HES. Clinically, the most frequent and most important manifestations of HES are thromboembolic events. Several immunological, serological, molecular, and cytogenetic markers are available to establish the nature of the underlying condition and thus to define the variant of HE and HES. Independent of the underlying condition, patients with established HES should be considered for early therapeutic intervention. In those with secondary HES, treatment of the underlying disease is usually effective. In some of these patients, IL-5-targeting antibodies can control HE. In myeloid neoplasms harboring mutated variants of *PDGFRA* or *PDGFRB*, imatinib or other PDGFR-targeting TKI are usually effective to control HE and to avoid HES-related organ damage. Our increasing knowledge about the etiology of HE, and the development of more specific markers and therapeutic approaches, should markedly improve diagnosis, management, and prognosis of patients with eosinophil disorders.

## References

[1] Gleich GJ (2000). Mechanisms of eosinophil-associated inflammation. J Allergy Clin Immunol.

[2] Kita H (2011). Eosinophils: multifaceted biological properties and roles in health and disease. Immunol Rev.

[3] Weller PF, Spencer LA (2017). Functions of tissue-resident eosinophils. Nat Rev Immunol.

[4] Klion AD, Ackerman SJ, Bochner BS (2020). Contributions of eosinophils to human health and disease. Annu Rev Pathol.

[5] Jacobsen EA, Jackson DJ, Heffler E, Mathur SK, Bredenoord AJ, Pavord ID, Akuthota P, Roufosse F, Rothenberg ME (2021). Eosinophil knockout humans: uncovering the role of eosinophils through eosinophil-directed biological therapies. Annu Rev Immunol.

[6] Akuthota P, Weller PF (2015). Spectrum of eosinophilic end-organ manifestations. Immunol Allergy Clin N Am.

[7] Curtis C, Ogbogu P (2016). Hypereosinophilic syndrome. Clin Rev Allergy Immunol.

[8] Simon D, Simon HU (2007). Eosinophilic disorders. J Allergy Clin Immunol.

[9] Simon HU, Rothenberg ME, Bochner BS, Weller PF, Wardlaw AJ, Wechsler ME, Rosenwasser LJ, Roufosse F, Gleich GJ, Klion AD (2010). Refining the definition of hypereosinophilic syndrome. J Allergy Clin Immunol.

[10] Valent P, Gleich GJ, Reiter A, Roufosse F, Weller PF, Hellmann A, Metzgeroth G, Leiferman KM, Arock M, Sotlar K, Butterfield JH, Cerny-Reiterer S, Mayerhofer M, Vandenberghe P, Haferlach T, Bochner BS, Gotlib J, Horny HP, Simon HU, Klion AD (2012). Pathogenesis and classification of eosinophil disorders: a review of recent developments in the field. Expert Rev Hematol.

[11] Valent P, Klion A, Horny HP, Roufosse F, Gotlib J, Weller PF, Hellmann A, Metzgeroth G, Leiferman KM, Arock M, Butterfield JH, Sperr WR, Sotlar K, Vandenberghe P, Haferlach T, Simon HU, Reiter A, Gleich GJ (2012). Contemporary consensus on criteria and classification of eosinophil disorders and related syndromes. J Allergy Clin Immunol.

[12] Kargili A, Bavbek N, Kaya A, Koşar A, Karaaslan Y (2004). Eosinophilia in rheumatologic diseases: a prospective study of 1000 cases. Rheumatol Int.

[13] Nutman TB (2007). Evaluation and differential diagnosis of marked, persistent eosinophilia. Immunol Allergy Clin N Am.

[14] Bain BJ, Fletcher SH (2007). Chronic eosinophilic leukemias and the myeloproliferative variant of the hypereosinophilic syndrome. Immunol Allergy Clin N Am.

[15] Valent P (2009). Pathogenesis, classification, and therapy of eosinophilia and eosinophil disorders. Blood Rev.

[16] Reiter A, Gotlib J (2017). Myeloid neoplasms with eosinophilia. Blood.

[17] Bain BJ (2010). Review: eosinophils and eosinophilic leukemia. Clin Adv Hematol Oncol.

[18] Gotlib J (2017). World Health Organization-defined eosinophilic disorders: 2017 update on diagnosis, risk stratification, and management. Am J Hematol.

[19] Mattis DM, Wang SA, Lu CM (2020). Contemporary classification and diagnostic evaluation of hypereosinophilia. Am J Clin Pathol.

[20] Leary AG, Ogawa M (1984). Identification of pure and mixed basophil colonies in culture of human peripheral blood and marrow cells. Blood.

[21] Denburg JA, Telizyn S, Messner H, Lim B, Jamal N, Ackerman SJ, Gleich GJ, Bienenstock J (1985). Heterogeneity of human peripheral blood eosinophil-type colonies: evidence for a common basophil-eosinophil progenitor. Blood.

[22] Shalit M, Sekhsaria S, Mauhorter S, Mahanti S, Malech HL (1996). Early commitment to the eosinophil lineage by cultured human peripheral blood CD34+ cells: messenger RNA analysis. J Allergy Clin Immunol.

[23] Denburg JA (1998). Hemopoietic progenitors and cytokines in allergic inflammation. Allergy.

[24] Linden M, Svensson C, Andersson M, Greiff L, Andersson E, Denburg JA, Persson CG (1999). Circulating eosinophil/basophil progenitors and nasal mucosal cytokines in seasonal allergic rhinitis. Allergy.

[25] Denburg JA, Richardson M, Telizyn S, Bienenstock J (1983). Basophil/mast cell precursors in human peripheral blood. Blood.

[26] Clutterbuck E, Shields JG, Gordon J, Smith SH, Boyd A, Callard RE, Campbell HD, Young IG, Sanderson CJ (1987). Recombinant human interleukin 5 is an eosinophil differentiation factor but has no activity in standard human B cell growth factor assays. Eur J Immunol.

[27] Saito H, Hatake K, Dvorak AM, Leiferman KM, Donnenberg AD, Arai N, Ishizaka K, Ishizaka T (1988). Selective differentiation and proliferation of hematopoietic cells induced by recombinant human interleukins. Proc Natl Acad Sci U S A.

[28] Valent P, Schmidt G, Besemer J, Mayer P, Zenke G, Liehl E, Hinterberger W, Lechner K, Maurer D, Bettelheim P (1989). Interleukin-3 is a differentiation factor for human basophils. Blood.

[29] Lopez AF, Vadas MA, Woodcock JM, Milton SE, Lewis A, Elliott MJ, Gillis D, Ireland R, Olwell E, Park LS (1991). Interleukin-5, interleukin-3, and granulocyte-macrophage colony-stimulating factor cross-compete for binding to cell surface receptors on human eosinophils. J Biol Chem.

[30] Lopez AF, Elliott MJ, Woodcock J, Vadas MA (1992). GM-CSF, IL-3 and IL-5: cross-competition on human haemopoietic cells. Immunol Today.

[31] Yoshimura-Uchiyama C, Yamaguchi M, Nagase H, Matsushima K, Igarashi T, Iwata T, Yamamoto K, Hirai K (2003). Changing expression of IL-3 and IL-5 receptors in cultured human eosinophils. Biochem Biophys Res Commun.

[32] Wang JM, Rambaldi A, Biondi A, Chen ZG, Sanderson CJ, Mantovani A (1989). Recombinant human interleukin 5 is a selective eosinophil chemoattractant. Eur J Immunol.

[33] Ebisawa M, Liu MC, Yamada T, Kato M, Lichtenstein LM, Bochner BS, Schleimer RP (1994). Eosinophil transendothelial migration induced by cytokines. II. Potentiation of eosinophil transendothelial migration by eosinophil-active cytokines. J Immunol.

[34] Simon HU, Yousefi S, Schranz C, Schapowal A, Bachert C, Blaser K (1997). Direct demonstration of delayed eosinophil apoptosis as a mechanism causing tissue eosinophilia. J Immunol.

[35] Bach MK, Brashler JR, Stout BK, Johnson HG, Sanders ME, Lin AH, Gorman RR, Bienkowski MJ, Ishizaka T (1992). Activation of human eosinophils by platelet-derived growth factor. Int Arch Allergy Immunol.

[36] Horie S, Okubo Y, Hossain M, Sato E, Nomura H, Koyama S, Suzuki J, Isobe M, Sekiguchi M (1997). Interleukin-13 but not interleukin-4 prolongs eosinophil survival and induces eosinophil chemotaxis. Intern Med.

[37] Noga O, Englmann C, Hanf G, Grützkau A, Guhl S, Kunkel G (2002). Activation of the specific neurotrophin receptors TrkA, TrkB and TrkC influences the function of eosinophils. Clin Exp Allergy.

[38] Rot A, Krieger M, Brunner T, Bischoff SC, Schall TJ, Dahinden CA (1992). RANTES and macrophage inflammatory protein 1 alpha induce the migration and activation of normal human eosinophil granulocytes. J Exp Med.

[39] Dahinden CA, Geiser T, Brunner T, von Tscharner V, Caput D, Ferrara P, Minty A, Baggiolini M (1994). Monocyte chemotactic protein 3 is a most effective basophil- and eosinophil-activating chemokine. J Exp Med.

[40] Noso N, Proost P, Van Damme J, Schröder JM (1994). Human monocyte chemotactic proteins-2 and 3 (MCP-2 and MCP-3) attract human eosinophils and desensitize the chemotactic responses towards RANTES. Biochem Biophys Res Commun.

[41] Ponath PD, Qin S, Ringler DJ, Clark-Lewis I, Wang J, Kassam N, Smith H, Shi X, Gonzalo JA, Newman W, Gutierrez-Ramos JC, Mackay CR (1996). Cloning of the human eosinophil chemoattractant, eotaxin. Expression, receptor binding, and functional properties suggest a mechanism for the selective recruitment of eosinophils. J Clin Invest.

[42] Rothenberg ME, Ownbey R, Mehlhop PD, Loiselle PM, van de Rijn M, Bonventre JV, Oettgen HC, Leder P, Luster AD (1996). Eotaxin triggers eosinophil-selective chemotaxis and calcium flux via a distinct receptor and induces pulmonary eosinophilia in the presence of interleukin 5 in mice. Mol Med.

[43] Okada S, Kita H, George TJ, Gleich GJ, Leiferman KM (1997). Transmigration of eosinophils through basement membrane components in vitro: synergistic effects of platelet-activating factor and eosinophil-active cytokines. Am J Respir Cell Mol Biol.

[44] Petering H, Götze O, Kimmig D, Smolarski R, Kapp A, Elsner J (1999). The biologic role of interleukin-8: functional analysis and expression of CXCR1 and CXCR2 on human eosinophils. Blood.

[45] Bochner BS, Bickel CA, Taylor ML, MacGlashan DW, Gray PW, Raport CJ, Godiska R (1999). Macrophage-derived chemokine induces human eosinophil chemotaxis in a CC chemokine receptor 3- and CC chemokine receptor 4-independent manner. J Allergy Clin Immunol.

[46] White JR, Lee JM, Dede K, Imburgia CS, Jurewicz AJ, Chan G, Fornwald JA, Dhanak D, Christmann LT, Darcy MG, Widdowson KL, Foley JJ, Schmidt DB, Sarau HM (2000). Identification of potent, selective non-peptide CC chemokine receptor-3 antagonist that inhibits eotaxin-, eotaxin-2-, and monocyte chemotactic protein-4-induced eosinophil migration. J Biol Chem.

[47] Menzies-Gow A, Ying S, Sabroe I, Stubbs VL, Soler D, Williams TJ, Kay AB (2002). Eotaxin (CCL11) and eotaxin-2 (CCL24) induce recruitment of eosinophils, basophils, neutrophils, and macrophages as well as features of early- and late-phase allergic reactions following cutaneous injection in human atopic and nonatopic volunteers. J Immunol.

[48] Sadovnik I, Lierman E, Peter B, Herrmann H, Suppan V, Stefanzl G, Haas O, Lion T, Pickl W, Cools J, Vandenberghe P, Valent P (2014). Identification of Ponatinib as a potent inhibitor of growth, migration, and activation of neoplastic eosinophils carrying FIP1L1-PDGFRA. Exp Hematol.

[49] Youngblood BA, Leung J, Falahati R, Williams J, Schanin J, Brock EC, Singh B, Chang AT, O’Sullivan JA, Schleimer RP, Tomasevic N, Bebbington CR, Bochner BS (2020). Discovery, function, and therapeutic targeting of Siglec-8. Cells.

[50] Munitz A, Levi-Schaffer F (2007). Inhibitory receptors on eosinophils: a direct hit to a possible Achilles heel?. J Allergy Clin Immunol.

[51] Sillaber C, Geissler K, Scherrer R, Kaltenbrunner R, Bettelheim P, Lechner K, Valent P (1992). Type beta transforming growth factors promote interleukin-3 (IL-3)-dependent differentiation of human basophils but inhibit IL-3-dependent differentiation of human eosinophils. Blood.

[52] Atsuta J, Fujisawa T, Iguchi K, Terada A, Kamiya H, Sakurai M (1995). Inhibitory effect of transforming growth factor beta 1 on cytokine-enhanced eosinophil survival and degranulation. Int Arch Allergy Immunol.

[53] de Bruin AM, Buitenhuis M, van der Sluijs KF, van Gisbergen KP, Boon L, Nolte MA (2010). Eosinophil differentiation in the bone marrow is inhibited by T cell-derived IFN-gamma. Blood.

[54] Alam R, Forsythe P, Stafford S, Fukuda Y (1994). Transforming growth factor beta abrogates the effects of hematopoietins on eosinophils and induces their apoptosis. J Exp Med.

[55] Stoeckle C, Geering B, Yousefi S, Rožman S, Andina N, Benarafa C, Simon HU (2016). RhoH is a negative regulator of eosinophilopoiesis. Cell Death Differ.

[56] Park CS, Choi EN, Kim JS, Choi YS, Rhim TY, Chang HS, Chung IY (2005). Interferon-gamma inhibits in vitro mobilization of eosinophils by interleukin-5. Int Arch Allergy Immunol.

[57] Peterson AP, Altman LC, Hill JS, Gosney K, Kadin ME (1981). Glucocorticoid receptors in normal human eosinophils: comparison with neutrophils. J Allergy Clin Immunol.

[58] Prin L, Lefebvre P, Gruart V, Capron M, Storme L, Formstecher P, Loiseau S, Capron A (1989). Heterogeneity of human eosinophil glucocorticoid receptor expression in hypereosinophilic patients: absence of detectable receptor correlates with resistance to corticotherapy. Clin Exp Immunol.

[59] Valent P (1994). The phenotype of human eosinophils, basophils, and mast cells. J Allergy Clin Immunol.

[60] Bochner BS (2000). Systemic activation of basophils and eosinophils: markers and consequences. J Allergy Clin Immunol.

[61] Hartnell A, Moqbel R, Walsh GM, Bradley B, Kay AB (1990). Fc gamma and CD11/CD18 receptor expression on normal density and low density human eosinophils. Immunology.

[62] Bochner BS, Luscinskas FW, Gimbrone MA, Newman W, Sterbinsky SA, Derse-Anthony CP, Klunk D, Schleimer RP (1991). Adhesion of human basophils, eosinophils, and neutrophils to interleukin 1-activated human vascular endothelial cells: contributions of endothelial cell adhesion molecules. J Exp Med.

[63] Ebisawa M, Bochner BS, Georas SN, Schleimer RP (1992). Eosinophil transendothelial migration induced by cytokines. I. Role of endothelial and eosinophil adhesion molecules in IL-1 beta-induced transendothelial migration. J Immunol.

[64] Bochner BS, Schleimer RP (1994). The role of adhesion molecules in human eosinophil and basophil recruitment. J Allergy Clin Immunol.

[65] Wein M, Sterbinsky SA, Bickel CA, Schleimer RP, Bochner BS (1995). Comparison of human eosinophil and neutrophil ligands for P-selectin: ligands for P-selectin differ from those for E-selectin. Am J Respir Cell Mol Biol.

[66] Matsumoto K, Sterbinsky SA, Bickel CA, Zhou DF, Kovach NL, Bochner BS (1997). Regulation of alpha 4 integrin-mediated adhesion of human eosinophils to fibronectin and vascular cell adhesion molecule-1. J Allergy Clin Immunol.

[67] Grayson MH, Van der Vieren M, Sterbinsky SA, Gallatin WM, Hoffman PA, Staunton DE, Bochner BS (1998). alphadbeta2 integrin is expressed on human eosinophils and functions as an alternative ligand for vascular cell adhesion molecule 1 (VCAM-1). J Exp Med.

[68] Wardlaw AJ (2000). The role of adhesion in eosinophil function. Chem Immunol.

[69] Bochner BS, Schleimer RP (2001). Mast cells, basophils, and eosinophils: distinct but overlapping pathways for recruitment. Immunol Rev.

[70] Schleimer RP, Bochner BS (1994). The effects of glucocorticoids on human eosinophils. J Allergy Clin Immunol.

[71] Kaiser J, Bickel CA, Bochner BS, Schleimer RP (1993). The effects of the potent glucocorticoid budesonide on adhesion of eosinophils to human vascular endothelial cells and on endothelial expression of adhesion molecules. J Pharmacol Exp Ther.

[72] Nagase H, Miyamasu M, Yamaguchi M, Kawasaki H, Ohta K, Yamamoto K, Morita Y, Hirai K (2000). Glucocorticoids preferentially upregulate functional CXCR4 expression in eosinophils. J Allergy Clin Immunol.

[73] Daffern PJ, Pfeifer PH, Ember JA, Hugli TE (1995). C3a is a chemotaxin for human eosinophils but not for neutrophils. I. C3a stimulation of neutrophils is secondary to eosinophil activation. J Exp Med.

[74] Aizawa H, Plitt J, Bochner BS (2002). Human eosinophils express two Siglec-8 splice variants. J Allergy Clin Immunol.

[75] Nagase H, Okugawa S, Ota Y, Yamaguchi M, Tomizawa H, Matsushima K, Ohta K, Yamamoto K, Hirai K (2003). Expression and function of Toll-like receptors in eosinophils: activation by Toll-like receptor 7 ligand. J Immunol.

[76] Hudson SA, Bovin NV, Schnaar RL, Crocker PR, Bochner BS (2009). Eosinophil-selective binding and proapoptotic effect in vitro of a synthetic Siglec-8 ligand, polymeric 6'-sulfated sialyl Lewis x. J Pharmacol Exp Ther.

[77] Bochner BS, Gleich GJ (2010). What targeting eosinophils has taught us about their role in diseases. J Allergy Clin Immunol.

[78] Hudson SA, Herrmann H, Du J, Cox P, Haddad EB, Butler B, Crocker PR, Ackerman SJ, Valent P, Bochner BS (2011). Developmental, malignancy-related and cross-species analysis of eosinophil, mast cell and basophil Siglec-8 expression. J Clin Immunol.

[79] Simon HU, Plötz S, Simon D, Seitzer U, Braathen LR, Menz G, Straumann A, Dummer R, Levi-Schaffer F (2003). Interleukin-2 primes eosinophil degranulation in hypereosinophilia and Wells’ syndrome. Eur J Immunol.

[80] Czech W, Krutmann J, Budnik A, Schöpf E, Kapp A (1993). Induction of intercellular adhesion molecule 1 (ICAM-1) expression in normal human eosinophils by inflammatory cytokines. J Invest Dermatol.

[81] Moqbel R, Levi-Schaffer F, Kay AB (1994). Cytokine generation by eosinophils. J Allergy Clin Immunol.

[82] Kay AB, Barata L, Meng Q, Durham SR, Ying S (1997). Eosinophils and eosinophil-associated cytokines in allergic inflammation. Int Arch Allergy Immunol.

[83] Spencer LA, Szela CT, Perez SA, Kirchhoffer CL, Neves JS, Radke AL, Weller PF (2009). Human eosinophils constitutively express multiple Th1, Th2 and immunoregulatory cytokines that are secreted rapidly and differentially. J Leukoc Biol.

[84] Davoine F, Lacy P (2014). Eosinophil cytokines, chemokines, and growth factors: emerging roles in immunity. Front Immunol.

[85] Kanda A, Yasutaka Y, Van Bui D, Suzuki K, Sawada S, Kobayashi Y, Asako M, Iwai H (2020). Multiple biological aspects of eosinophils in host defense, eosinophil-associated diseases, immunoregulation, and homeostasis: is their role beneficial, detrimental, regulator, or bystander?. Biol Pharm Bull.

[86] Lehrer RI, Szklarek D, Barton A, Ganz T, Hamann KJ, Gleich GJ (1989). Antibacterial properties of eosinophil major basic protein and eosinophil cationic protein. J Immunol.

[87] Hamann KJ, Barker RL, Ten RM, Gleich GJ (1991). The molecular biology of eosinophil granule proteins. Int Arch Allergy Appl Immunol.

[88] Yousefi S, Gold JA, Andina N, Lee JJ, Kelly AM, Kozlowski E, Schmid I, Straumann A, Reichenbach J, Gleich GJ, Simon HU (2008). Catapult-like release of mitochondrial DNA by eosinophils contributes to antibacterial defense. Nat Med.

[89] Persson EK, Verstraete K, Heyndrickx I, Gevaert E, Aegerter H, Percier JM, Deswarte K, Verschueren KHG, Dansercoer A, Gras D, Chanez P, Bachert C, Gonçalves A, Van Gorp H, De Haard H, Blanchetot C, Saunders M, Hammad H, Savvides SN, Lambrecht BN (2019). Protein crystallization promotes type 2 immunity and is reversible by antibody treatment. Science.

[90] Swartz JM, Byström J, Dyer KD, Nitto T, Wynn TA, Rosenberg HF (2004). Plasminogen activator inhibitor-2 (PAI-2) in eosinophilic leukocytes. J Leukoc Biol.

[91] Noguchi H, Kephart GM, Colby TV, Gleich GJ (1992). Tissue eosinophilia and eosinophil degranulation in syndromes associated with fibrosis. Am J Pathol.

[92] Neves JS, Weller PF (2009). Functional extracellular eosinophil granules: novel implications in eosinophil immunobiology. Curr Opin Immunol.

[93] Simon D, Hoesli S, Roth N, Staedler S, Yousefi S, Simon HU (2011). Eosinophil extracellular DNA traps in skin diseases. J Allergy Clin Immunol.

[94] Sastre B, Rodrigo-Muñoz JM, Garcia-Sanchez DA, Cañas JA, Del Pozo V (2018). Eosinophils: old players in a new game. J Investig Allergol Clin Immunol.

[95] Chusid MJ, Dale DC, West BC, Wolff SM (1975). The hypereosinophilic syndrome: analysis of fourteen cases with review of the literature. Medicine.

[96] Kato M, Kephart GM, Talley NJ, Wagner JM, Sarr MG, Bonno M, McGovern TW, Gleich GJ (1998). Eosinophil infiltration and degranulation in normal human tissue. Anat Rec.

[97] Soragni A, Yousefi S, Stoeckle C, Soriaga AB, Sawaya MR, Kozlowski E, Schmid I, Radonjic-Hoesli S, Boutet S, Williams GJ, Messerschmidt M, Seibert MM, Cascio D, Zatsepin NA, Burghammer M, Riekel C, Colletier JP, Riek R, Eisenberg DS, Simon HU (2015). Toxicity of eosinophil MBP is repressed by intracellular crystallization and promoted by extracellular aggregation. Mol Cell.

[98] Roufosse F, Schandené L, Sibille C, Willard-Gallo K, Kennes B, Efira A, Goldman M, Cogan E (2000). Clonal Th2 lymphocytes in patients with the idiopathic hypereosinophilic syndrome. Br J Haematol.

[99] Roufosse F, Cogan E, Goldman M (2003). The hypereosinophilic syndrome revisited. Annu Rev Med.

[100] Roufosse F, Cogan E, Goldman M (2007). Lymphocytic variant hypereosinophilic syndromes. Immunol Allergy Clin N Am.

[101] Simon HU, Plötz SG, Dummer R, Blaser K (1999). Abnormal clones of T cells producing interleukin-5 in idiopathic eosinophilia. N Engl J Med.

[102] Hardy WR, Anderson RE (1968). The hypereosinophilic syndromes. Ann Intern Med.

[103] Wilkins HJ, Crane MM, Copeland K, Williams WV (2005). Hypereosinophilic syndrome: an update. Am J Hematol.

[104] Weller PF, Bubley GJ (1994). The idiopathic hypereosinophilic syndrome. Blood.

[105] Apperley JF, Gardembas M, Melo JV, Russell-Jones R, Bain BJ, Baxter EJ, Chase A, Chessells JM, Colombat M, Dearden CE, Dimitrijevic S, Mahon FX, Marin D, Nikolova Z, Olavarria E, Silberman S, Schultheis B, Cross NCP, Goldman JM (2002). Response to imatinib mesylate in patients with chronic myeloproliferative diseases with rearrangements of the platelet-derived growth factor receptor beta. N Engl J Med.

[106] Cools J, DeAngelo DJ, Gotlib J, Stover EH, Legare RD, Cortes J, Kutok J, Clark J, Galinsky I, Griffin JD, Cross NCP, Tefferi A, Malone J, Alam R, Schrier SL, Schmid J, Rose M, Vandenberghe P, Verhoef G, Boogaerts M, Wlodarska I, Kantarjian H, Marynen P, Coutre SE, Stone R, Gilliland DG (2003). A tyrosine kinase created by fusion of the PDGFRA and FIP1L1 genes as a therapeutic target of imatinib in idiopathic hypereosinophilic syndrome. N Engl J Med.

[107] Metzgeroth G, Walz C, Erben P, Popp H, Schmitt-Graeff A, Haferlach C, Fabarius A, Schnittger S, Grimwade D, Cross NCP, Hehlmann R, Hochhaus A, Reiter A (2008). Safety and efficacy of imatinib in chronic eosinophilic leukaemia and hypereosinophilic syndrome: a phase-II study. Br J Haematol.

[108] Krauth MT, Binder T, Ohler L, Jäger U, Valent P (2008). Improvement of cardiac function, mitral regurgitation and pulmonary hypertension in a patient with chronic eosinophilic leukemia (CEL) after low dose imatinib therapy. Leuk Res.

[109] Klion AD (2015). Eosinophilia: a pragmatic approach to diagnosis and treatment. Hematology Am Soc Hematol Educ Program.

[110] Khoury P, Bochner BS (2018). Consultation for elevated blood eosinophils: clinical presentations, high value diagnostic tests, and treatment options. J Allergy Clin Immunol Pract.

[111] Gleich GJ, Schroeter AL, Marcoux JP, Sachs MI, O’Connell EJ, Kohler PF (1984). Episodic angioedema associated with eosinophilia. N Engl J Med.

[112] Butterfield JH, Leiferman KM, Abrams J, Silver JE, Bower J, Gonchoroff N, Gleich GJ (1992). Elevated serum levels of interleukin-5 in patients with the syndrome of episodic angioedema and eosinophilia. Blood.

[113] Khoury P, Herold J, Alpaugh A, Dinerman E, Holland-Thomas N, Stoddard J, Gurprasad S, Maric I, Simakova O, Schwartz LB, Fong J, Lee CC, Xi L, Wang Z, Raffeld M, Klion AD (2015). Episodic angioedema with eosinophilia (Gleich syndrome) is a multilineage cell cycling disorder. Haematologica.

[114] Gross WL (2002). Churg-Strauss syndrome: update on recent developments. Curr Opin Rheumatol.

[115] Keogh KA, Specks U (2006). Churg-Strauss syndrome. Semin Respir Crit Care Med.

[116] Pagnoux C, Guillevin L (2010). Churg-Strauss syndrome: evidence for disease subtypes?. Curr Opin Rheumatol.

[117] Silver RM (1992). Eosinophilia-myalgia syndrome, toxic-oil syndrome, and diffuse fasciitis with eosinophilia. Curr Opin Rheumatol.

[118] Kaufman LD, Krupp LB (1995). Eosinophilia-myalgia syndrome, toxic-oil syndrome, and diffuse fasciitis with eosinophilia. Curr Opin Rheumatol.

[119] Belongia EA, Gleich GJ (1996). The eosinophilia-myalgia syndrome revisited. J Rheumatol.

[120] Klion AD, Bochner BS, Gleich GJ, Nutman TB, Rothenberg ME, Simon HU, Wechsler ME, Weller PF (2006). Approaches to the treatment of hypereosinophilic syndromes: a workshop summary report. J Allergy Clin Immunol.

[121] Ogbogu PU, Bochner BS, Butterfield JH, Gleich GJ, Huss-Marp J, Kahn JE, Leiferman KM, Nutman TB, Pfab F, Ring J, Rothenberg ME, Roufosse F, Sajous MH, Sheikh J, Simon D, Simon HU, Stein ML, Wardlaw A, Weller PF, Klion AD (2009). Hypereosinophilic syndrome: a multicenter, retrospective analysis of clinical characteristics and response to therapy. J Allergy Clin Immunol.

[122] Simon HU, Cools J (2007). Novel approaches to therapy of hypereosinophilic syndromes. Immunol Allergy Clin N Am.

[123] Roufosse F, Weller PF (2010). Practical approach to the patient with hypereosinophilia. J Allergy Clin Immunol.

[124] Klion A (2018). Hypereosinophilic syndrome: approach to treatment in the era of precision medicine. Hematology Am Soc Hematol Educ Program.

[125] Plötz SG, Simon HU, Darsow U, Simon D, Vassina E, Yousefi S, Hein R, Smith T, Behrendt H, Ring J (2003). Use of an anti-interleukin-5 antibody in the hypereosinophilic syndrome with eosinophilic dermatitis. N Engl J Med.

[126] Rothenberg ME, Klion AD, Roufosse FE, Kahn JE, Weller PF, Simon HU, Schwartz LB, Rosenwasser LJ, Ring J, Griffin EF, Haig AE, Frewer PIH, Parkin JM, Gleich GJ (2008). Treatment of patients with the hypereosinophilic syndrome with mepolizumab. N Engl J Med.

[127] Roufosse F, de Lavareille A, Schandené L, Cogan E, Georgelas A, Wagner L, Xi L, Raffeld M, Goldman M, Gleich GJ, Klion A (2010). Mepolizumab as a corticosteroid-sparing agent in lymphocytic variant hypereosinophilic syndrome. J Allergy Clin Immunol.

[128] Radonjic-Hoesli S, Valent P, Klion AD, Wechsler ME, Simon HU (2015). Novel targeted therapies for eosinophil-associated diseases and allergy. Annu Rev Pharmacol Toxicol.

[129] Roufosse F, Kahn JE, Rothenberg ME, Wardlaw AJ, Klion AD, Kirby SY, Gilson MJ, Bentley JH, Bradford ES, Yancey SW, Steinfeld J, Gleich GJ, group HESMs (2020). Efficacy and safety of mepolizumab in hypereosinophilic syndrome: A phase III, randomized, placebo-controlled trial. J Allergy Clin Immunol.

[130] Jovanovic JV, Score J, Waghorn K, Cilloni D, Gottardi E, Metzgeroth G, Erben P, Popp H, Walz C, Hochhaus A, Roche-Lestienne C, Preudhomme C, Solomon E, Apperley J, Rondoni M, Ottaviani E, Martinelli G, Brito-Babapulle F, Saglio G, Hehlmann R, Cross NCP, Reiter A, Grimwade D (2007). Low-dose imatinib mesylate leads to rapid induction of major molecular responses and achievement of complete molecular remission in FIP1L1-PDGFRA-positive chronic eosinophilic leukemia. Blood.

[131] Reiter A, Grimwade D, Cross NC (2007). Diagnostic and therapeutic management of eosinophilia-associated chronic myeloproliferative disorders. Haematologica.

[132] Gotlib J, Cools J (2008). Five years since the discovery of FIP1L1-PDGFRA: what we have learned about the fusion and other molecularly defined eosinophilias. Leukemia.

[133] Simon D, Salemi S, Yousefi S, Simon HU (2008). Primary resistance to imatinib in Fip1-like 1-platelet-derived growth factor receptor alpha-positive eosinophilic leukemia. J Allergy Clin Immunol.

[134] Salemi S, Yousefi S, Simon D, Schmid I, Moretti L, Scapozza L, Simon HU (2009). A novel FIP1L1-PDGFRA mutant destabilizing the inactive conformation of the kinase domain in chronic eosinophilic leukemia/hypereosinophilic syndrome. Allergy.

